# Genome-Wide Identification of *GmARF9b*/*GmARF2a* Negatively Regulate Root Growth in Soybean

**DOI:** 10.3390/ijms26104547

**Published:** 2025-05-09

**Authors:** Di Li, Tianle Miao, Hong Liao, Yongjia Zhong

**Affiliations:** Root Biology Center, College of Resources and Environment, Fujian Agriculture and Forestry University, Fuzhou 350002, China; m17600702609@outlook.com (D.L.); 18699205447@163.com (T.M.)

**Keywords:** *GmARFs*, root, gene family, domain analysis, expression profiling, soybean

## Abstract

Auxin is the most important phytohormone, regulating root growth and development in plants. ARFs function as central regulators in auxin signaling in model plants, and the functions of ARF activators have been widely investigated, while the functions of ARF repressors remain elusive. In this study, we investigated the family of *GmARFs* in soybean through a genome-wide expression pattern and functional study of roots highly expressing *ARFs*. In total, we identified 59 *GmARF* members in the soybean genome. GmARFs harbor canonical B3 DNA-binding (B3), ARF (Aux rep), and PB1 functional protein domains. We identified two potential repressor genes, *GmARF9b* (*Glyma.03G070500*) and *GmARF2a* (*Glyma.05G200800*), which are specifically or highly expressed in the roots. Histochemical staining suggested that both genes are primarily expressed in the stele, root tips, and lateral root primordia. Subcellular localization analysis showed they were mainly localized in the nucleus. Overexpression of *GmARF9b* and *GmARF2a* significantly inhibited root growth using a transgenic hairy root assay. Root section analysis further suggested that *GmARF9b* and *GmARF2a* negatively regulated cortical cell layers and the longitudinal cell length of roots, thereby modulating root growth. Overall, this study has preliminarily identified two *GmARFs* that inhibit root growth in soybean. This discovery has deepened our understanding of the functions of ARFs in root development and has provided guidance for breeding crop roots with improved nutrient use efficiency.

## 1. Introductions

It is known that auxin is the most essential phytohormone, regulating root growth and development, including vascular tissue differentiation, root primordium formation, cell division, and differentiation [1,2]. Auxin regulates plant growth and development through specific signaling transduction, among which the intracellular Aux/IAA-TIR1-ARF pathway is critical for auxin signaling transduction. This pathway consists of three components, Aux/IAAs, auxin response factors (ARFs), and auxin receptors (transport inhibitor resistant 1/auxin signaling F-box, TIR1/AFB). When the exogenous auxin concentration is low, protein domain I of Aux/IAA interacts with the ARF PB1 domain, which inhibits the expression of downstream genes by ARFs. When the auxin concentration is high, the TIR1/AFB receptor interacts with Aux/IAAs, leading to ubiquitination of the Aux/IAAs protein and thus protein degradation through the 26S proteasome pathway. Thereby, it releases ARF transcription factors to induce the expression of auxin response genes [3]. Hence, ARFs are the key transcription factors in the auxin signaling pathway.

To date, numerous ARF (auxin response factor) genes have been identified across diverse plant species [4]. There are 22 ARFs, 25 ARFs, 59 ARFs, 22 AFRs, 25 ARFs, 23 ARFs, 25 ARFs, 19 ARFs, and 36 ARFs that have been identified in *Arabidopsis thaliana*, *Brassica oleracea* var. *capitata Linnaeus*, *Glycine max* (L.) *Merr.*, *Oryza sativa*, *Triticum aestivum*, *Sorghum bicolor*, *Vitis vinifera* L., and *Zea mays* [4,5,6,7,8]. Systematic analyses of ARF gene families across diverse plant species have enabled comprehensive functional characterization of ARF transcription factors in model organisms. Current research has demonstrated that auxin response factor (ARF) proteins typically consist of three evolutionarily conserved domains, the DNA-binding domain (DBD), the middle region (MR), and the protein C-terminal domain (CTD) [5]. The DNA-binding domain (DBD) of ARFs is B3 type DBD, which is the key domain responsible for DNA binding [9]. The middle region (MR) is a key non-conserved domain that determines the activation domain (AD) or repression domain (RD) of ARF. The C-terminal domain (CTD) shares similar amino acid sequences with the PB1 domain (motifs III/IV) of Aux/IAA proteins, and these two domains interact through these homologous regions to form a transcriptional complex [5].

Further functional studies reveal that ARF gene family members in diverse plant species finely regulate root development through the synergistic action of conserved structural domains and species-specific mechanisms. For instance, the *Arabidopsis* activator *AtARF5* maintains root apical meristems, the formation of lateral root primordia, and the lateral organ formation in the shoot apical meristem by regulating the expression of *AtPIN1*, *AtPIN3*, and *AtPIN7* [10,11,12]. *AtARF6* and *AtARF8* negatively regulate adventitious root (AR) formation, wherein overexpression of *AtARF8* leads to a reduction in free auxin content, which inhibits lateral root formation [5,13]. *AtARF7* and *AtARF19* are key genes redundantly regulating lateral root formation [14,15]. The rice transcriptional activator *OsARF1* can directly bind to the auxin response element (AuxRE) in the promoter of *OsCRL1*, activating the transcription of this gene in the initiation zones of adventitious roots (ARs) and lateral roots (LRs), thereby regulating root growth [16]. The maize transcriptional activator *ZmARF4* positively regulates the initiation and growth of lateral roots [17]. Taken together, ARF gene families of various crops are the key components that regulate root growth and development. However, most previous studies have predominantly focused on transcriptional activators, with limited attention to transcriptional suppressors and ARF genes in soybean.

In this study, we mainly focus on analyzing natural selection and the gene expression patterns of the ARF family in soybean to identify potential transcription repressors of *GmARFs* with specifically expressed in roots and further investigate the functions of the transcription repressor of *GmARFs* on root growth and development in soybean. Our study provides new insight into the functions of ARFs through understanding their underlying molecular mechanisms. It will also provide a theoretical basis for further research on the mining and utilization of superior allelic variant genes.

## 2. Results

### 2.1. Phylogenetic, Classification, and Gene Haplotype Analysis of GmARFs

**Figure 1:**
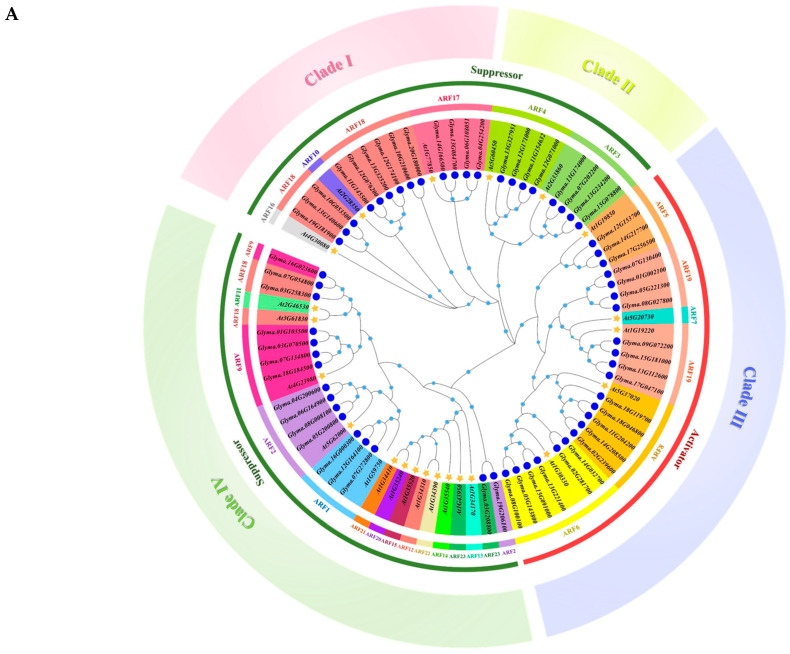

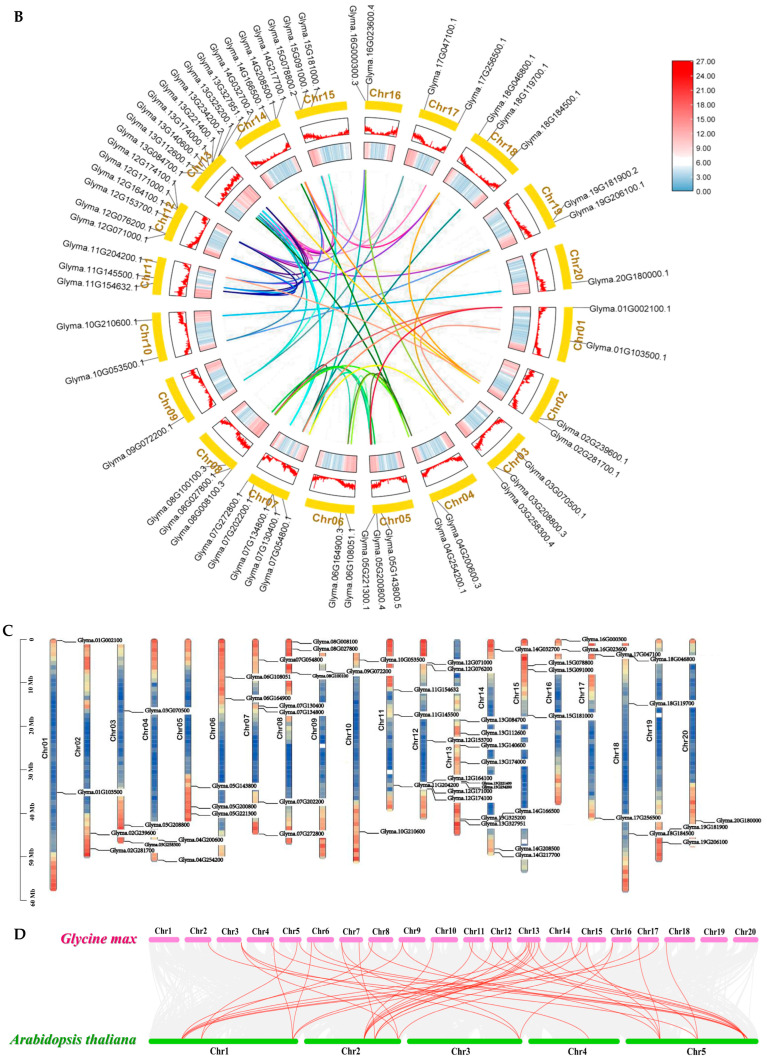
Phylogenetic tree and synteny analysis of ARF genes. (**A**) Phylogenetic tree of ARF proteins from *Glycine max* (Gm) and *Arabidopsis thaliana* (At). Yellow stars and blue circles indicate Arabidopsis and soybean, respectively. The light blue circle placed on the tree indicates the bootstrap value (display range: 0.51–1.00). The colored segments in the inner circle represent distinct subfamilies, while those in the outer circle denote different phylogenetic clades. (**B**) Distribution and collinearity of *GmARF* genes in soybean genome. Gray lines indicate synteny blocks in soybean genome, while lines of various colors indicate segmental duplicated ARF gene pairs in soybean. (**C**) Synteny analysis between soybean *GmARF* genes and Arabidopsis *AtARF* genes. Gray lines in the background indicate collinear blocks between soybean and *Arabidopsis* genomes, while red lines highlight the systematic gene pairs of *GmARFs* between soybean and Arabidopsis genomes. (**D**) The distribution of ARF genes on chromosomes. The chromosome number is indicated to the left of each chromosome.

To investigate the phylogenetic tree of GmARFs and determine the evolutionary relationship among each family member of GmARFs in soybean, the amino acid sequences of 23 AtARFs from *Arabidopsis thaliana* and 59 GmARF members from soybean (*Glycine max*) were analyzed. As shown in Figure 1, 23 Arabidopsis AtARFs and 59 soybean GmARF members were divided into two major groups. Among these, subclasses I, II, and III belong to group one, and subclass IV belongs to group two. Subclass I includes ARF10, ARF16, ARF17, and ARF18 subfamilies, with 3 members from *Arabidopsis thaliana* and 13 members from soybean. Subclass II includes the ARF3 and ARF4 subfamilies, with two members from *Arabidopsis thaliana* and eight members from soybean. Subclass III includes the ARF5, ARF6, ARF7, ARF8, and ARF19 subfamilies, with 5 members from *Arabidopsis thaliana* and 22 members from soybean. Subclass IV contains the ARF1, ARF2, ARF9, ARF11, ARF12, ARF13, ARF14, ARF15, ARF20, ARF21, ARF22, and ARF23 subfamilies, with a total of 13 members from *Arabidopsis thaliana* and 16 members from soybean. Previous studies have shown that *Arabidopsis* AtARFs can be divided into two types according to their functions: A-ARFs (activators), which include AtARF5, AtARF6, AtARF7, AtARF8, and AtARF19; and B-ARFs (repressors), which include AtARF1, AtARF2, AtARF3, AtARF4, AtARF9, AtARF17, AtARF18, and AtARF23. Therefore, subclasses I, II, and IV can be categorized as potential repressors, while subclass III can be categorized as activators.

Subclass and subfamily analyses showed that there are no soybean family members in the ARF10 and ARF16 subfamilies in Subclass I and no soybean family members in the ARF7 subfamily (Figure 1A). The ARF7 and ARF19 subfamilies were highly homologous, and the ARF6, ARF8, and ARF19 subfamilies belonged to the same large paraclade, sharing similar evolutionary processes and having close genetic relationships (Figure 1A). The evolutionary processes of each family member are complex. Additionally, there were no soybean family members In the ARF11, ARF21, ARF20, ARF15, ARF12, ARF22, ARF14, and ARF13 subfamilies. The ARF21, ARF20, ARF15, ARF12, ARF22, ARF14, ARF13, and ARF23 subfamilies belong to a major paralogue branch with a highly homologous relationship within members (Figure 1A). ARF11 and ARF18, as well as ARF2 and ARF23, constitute two pairs of subfamilies that exhibit highly homologous relationships within the same paralogue lineage.

Based on the differences in chromosome length, genome complexity, and genetic stability observed between the tetraploid soybean and the diploid *Arabidopsis thaliana*, the potential evolutionary relationship between ARFs (auxin response factors) from soybean and their homologous genes in *Arabidopsis thaliana* was further explored. Chromosomal distribution and collinearity analyses were conducted using Circos, MCScanX, and Tbtools. A total of 59 *GmARF* genes from soybean were located on 20 chromosomes (Figure 1B,C). More than 20% of these *GmARF* genes form a cluster on chromosome 13 (8, 13.56%) and chromosome 12 (6, 10.17%), while the remaining 76% of *GmARF* genes were distributed in other chromosomes, with each containing 1–4 *GmARF* genes. Among them, activator *GmARF* genes were found on all chromosomes except chr3, chr4, chr6, chr10, chr12, chr16, chr19, and chr20, and most of these genes were located at the ends of chromosomes. Forty percent of the repressor *GmARF* genes were distributed on chromosomes 7 (4, 10%), 12 (6, 15%), and 13 (6, 15%), while the rest of the genes were distributed on the other chromosomes.

Collinearity analysis revealed that there were 78 pairs of collinear genes within the *GmARF* gene family in soybean, encompassing 75 pairs belonging to the same subfamily and 3 pairs belonging to different subfamilies (Figure 1B). Among the members of the *GmARF2*, *GmARF3*, and *GmARF17* subfamilies, there were six pairs of collinear genes. Specifically, there were 1, 5, 2, and 24 pairs of collinear genes within the *GmARF1*, *GmARF4*, *GmARF9*, and *GmARF18* subfamilies, respectively. The activator subfamilies exhibit a certain degree of conservation among their gene members, as evidenced by the presence of 1, 3, 6, and 12 collinear gene pairs within the *GmARF5*, *GmARF6*, *GmARF8*, and *GmARF19* subfamilies, respectively (Figure 1B). These results suggest that genes within each subfamily might have similar functions and might participate in comparable regulatory networks. On the other hand, the repressor *GmARF23* shows collinearity with *Glyma.19G206100* from the repressor *GmARF2* subfamily; similarly, *Glyma.03G258300* and *Glyma.07G054800* from the repressor *GmARF18* subfamily exhibit collinear relationships with *Glyma.16G023600* from the repressor *GmARF9* subfamily. These findings indicate that, despite not belonging to the same subfamily, the aforementioned genes have the same conserved domains during evolution and may possess similar functions, indicating gene family expansion events of *GmARF18*, *GmARF2*, and *GmARF9*. Additionally, *Glyma.07G272800* from the repressor *GmARF1*, *Glyma.13G140600* from the repressor *GmARF18*, *Glyma.12G153700* from the activator *GmARF5*, and *Glyma.18G119700* from the activator *GmARF8* do not show collinear relationships with other genes, suggesting that these genes might evolve in different ways.

Collinearity analysis between soybean and Arabidopsis revealed 41 collinear gene pairs between *GmARFs* and *AtARFs* (Figure 1D). Based on the evolutionary tree, these gene pairs can be divided into two major categories: evolutionary gene families and non-homologous gene families. Within the homologous gene families, there are 26 collinear gene pairs, including four, four, three, three, and two pairs in the repressor *ARF2*, *ARF3*, *ARF4*, *ARF17*, and *ARF18* homologous gene families, respectively, and one, two, three, and four pairs in the activator *ARF5*, *ARF6*, *ARF8*, and *ARF19* homologous gene families, respectively. This indicates that members of homologous gene families have a similar arrangement pattern on their chromosomes, suggesting close genetic relationships or shared evolutionary histories. In contrast, there are 15 collinear gene pairs in the non-homologous gene families, suggesting a shift in the chromosomal locations after gene duplication.

### 2.2. Analysis of the Association Between GmARF Gene Haplotypes and Natural Selection

**Figure 2:**
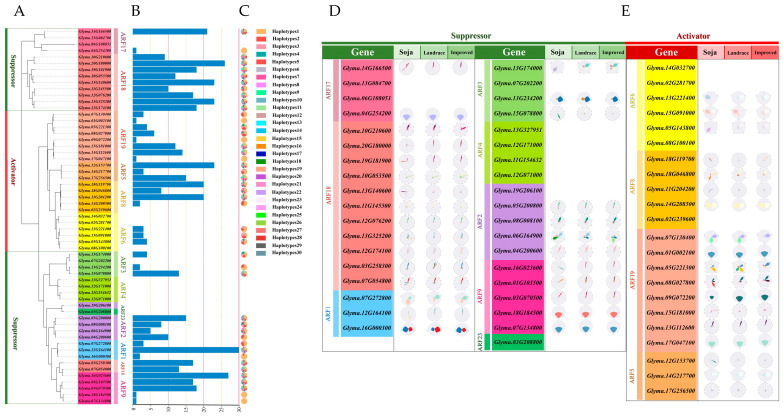
The phylogenetic relationship and haplotype analysis of *GmARFs* from *Glycine max* (Gm). (**A**) Phylogenetic tree of ARF proteins from *Glycine max* (Gm). Color blocks in different hues represent distinct subfamilies. (**B**) The phylogenetic tree of 59 *GmARFs*. (**C**) Haplotype analysis for *GmARF* genes and distribution of haplotype variations across each gene. The bar chart represents the number of haplotypes for each *GmARF* gene, and the pie chart illustrates the distribution of each haplotype. (**D**) Evolutionary distribution of suppressor *GmARFs*. Color blocks in different hues represent distinct subfamilies. (**E**) Evolutionary distribution of activator *GmARFs*. Petals of different colors represent different haplotypes. Color blocks in different hues represent distinct subfamilies.

Family and collinearity analyses indicate that the kinship among members of different subfamilies is relatively distinctive. To investigate the natural variation in the amino acid sequence of GmARFs, further haplotype analysis of the family genes was conducted (Figure 2). The results showed that the number of haplotypes for repressors *GmARF2/3/4/23* and activators *GmARF6/19* was lower than other *GmARFs*, with minimal haplotype differences among gene members within the subfamily (Figure 2B,C). The haplotype variation amplitudes are high within the families of the repressors *GmARF1/9/17/18* and activators *GmARF5/18*.

The *GmARFs* have undergone natural or artificial selection, because of the genetic variations during the transition from wild to cultivar, exhibiting a reduction in the number of alleles and reduced sequencing diversity. To further explore the genetic changes during the domestication of *GmARFs*, genetic structure maps were analyzed (Figure 2D,E). Specifically, the repressor subfamilies *GmARF1/2/3/17* and the activator subfamily *GmARF6* were selected during domestication. Furthermore, more than 60% of the members within each subfamily of repressors *GmARF9/18* and activators *GmARF5/8/19* possess two to five haplotypes that have undergone significant selection.

### 2.3. Gene Structure and Cis-Regulatory Element Analysis of GmARF Members

**Figure 3:**
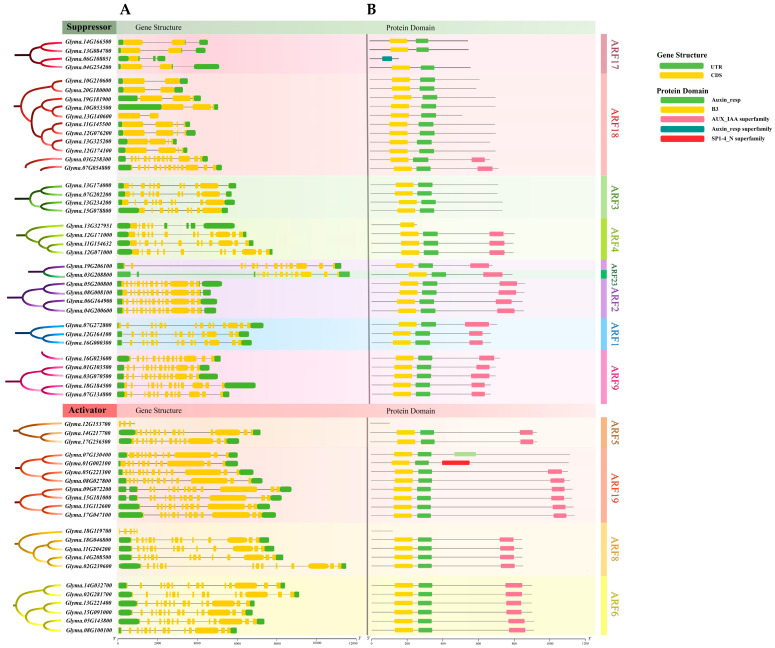
The gene structures of GmARFs. (**A**) The gene structures of the GmARFs include coding sequences (CDS) and untranslated regions (UTRs). (**B**) Distribution of protein domain of GmARF proteins.

To delve deeper into the evolutionary history of the GmARF family genes, TBtools was utilized to visualize the intron–exon structures and protein architectures of the coding sequences (Figure 3A). The results showed that the gene structures among different subfamilies vary greatly, yet the structures within each subfamily member were relatively conserved (Figure 3A). The repressor genes GmARF17 and GmARF18 have two–three exons and one–two introns, respectively. In contrast, the repressor genes GmARF1/2/3/4/9/23 harbor more complex gene structures, with 10–15 exons and 9–14 introns. The activator genes GmARF5/6/8/19 have 13–14 exons and 12–13 introns (Figure 3A).

Analysis of the conserved domains in GmARFs revealed that most of the 59 family members contained B3 and AUX_IAA domains (Figure 3B). Among them, the repressor genes GmARF1/2/4/9/23 and the activator genes GmARF5/6/8/19 had an AUX_IAA superfamily domain at the C′ terminus of their protein sequences, indicating that these subfamilies can directly interact with auxin/indole-3-acetic acid (Aux/IAA)-related proteins. In contrast, the repressor genes GmARF3/17/18 lacked a conserved domain at the C′ terminus of their protein, suggesting that these subfamilies have some degree of functional specialization and may require specific interactions with other proteins.

### 2.4. Whole-Genome Characterization of GmARF Genes in Soybean

To further investigate the physicochemical properties of the GmARF protein family, the amino acid sequences of this gene family were predicted using Expasy (https://web.expasy.org/protparam/, accessed on 20 November 2024) and NCBI (https://www.ncbi.nlm.nih.gov/, accessed on 20 November 2024). Overall, the physicochemical indices of most member proteins within each subfamily were similar. Notably, repressor-type GmARF subfamilies displayed significantly shorter amino acid sequences than activator-type GmARF subfamilies (Table 1). Conversely, the repressor subfamilies had higher isoelectric points.

Subfamily analysis shows that repressor-type GmARFs (GmARF2/4/23) and activator-type GmARFs (GmARF5/6/8/19) exhibit significantly longer amino acid sequences, higher molecular weights, and elevated instability indices, aliphatic indices (A.I.), and grand average hydropathicity (GRAVY) scores. Among them, repressors *GmARF2* (*Glyma.19G206100*) and *GmARF4* (*Glyma.13G327951*), as well as the activators *GmARF5* (*Glyma.12G153700*) and *GmARF8* (*Glyma.18G119700*), have amino acid lengths ranging from 109 to 677 and molecular weights between 12050.98 and 75645.75 Daltons. The repressors *GmARF1/3/9/17/18* have amino acid lengths ranging from 551 (*Glyma.13G084700*) to 739 (*Glyma.13G234200*), molecular weights between 18067.4 and 80922.02 Daltons, and instability indices between 48.05 (*Glyma.04G254200*) and 60.82 (*Glyma.12G164100*). The A.I. values range from 63.36 (*Glyma.13G084700*) to 76.46 (*Glyma.18G184500*). Subcellular localization predictions showed that most members are primarily located in the nucleus, suggesting that their potential transcriptional regulatory roles are likely mediated via nuclear functions. *GmARF17* is predicted to be primarily located in chloroplasts.

### 2.5. Conserved Motifs and Protein Structural Analysis of GmARF Genes

**Figure 4:**
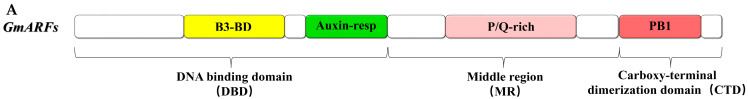

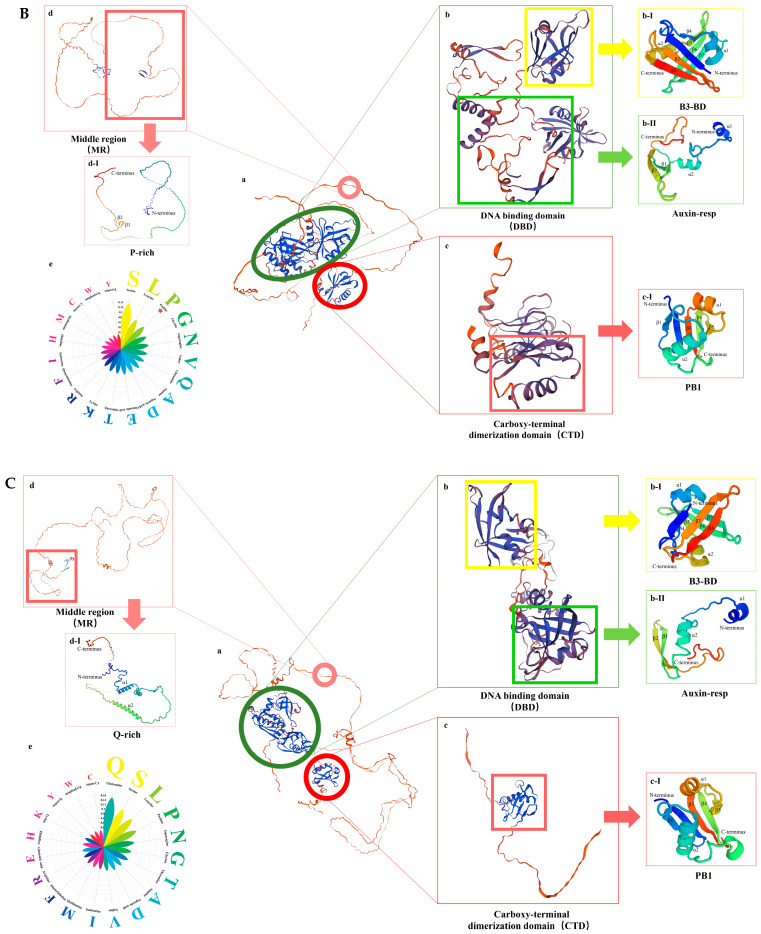
The structural analysis of GmARF proteins. (**A**) The distribution of conserved domains in GmARF proteins. (**B**) Three-dimensional structural diagrams of repressor protein. Each distinct colored block represents a unique protein domain. (**C**) Three-dimensional structural diagrams of activator protein Each distinct colored block represents a unique protein domain. (**B-a**) Construction of the overall 3D structural diagram using conserved amino acids from various repressor proteins; (**B-b**) 3D structural diagram of the DNA-binding domain: (**B-b-I**) B3 domain; (**B-b-II**) auxin-response domain; (**B-c**) 3D structural diagram of the carboxy-terminal dimerization domain binding region: (**B-c-I**) PB1 domain; (**B-d**) 3D structural diagram of the middle region binding domain: (**B-d-I**) structural diagram of core amino acids; (**B-e**) amino acid composition percentage diagram. (**C-a**) Construction of the overall 3D structural diagram using conserved amino acids from various activator proteins; (**C-b**) 3D structural diagram of the DNA-binding domain: (**C-b-I**) B3 domain; (**C-b-II**) auxin-response domain; (**C-c**) 3D structural diagram of the carboxy-terminal dimerization domain binding region: (**C-c-I**) PB1 domain; (**C-d**) 3D structural diagram of the middle region binding domain: (**C-d-I**) structural diagram of core amino acids; (**C-e**) amino acid composition percentage diagram.

ARFs (auxin response factors) are the key transcription factors in the auxin signaling pathway. They usually contain three domains, as illustrated in Figure 4A: the DNA-binding domain (DBD), the middle region (MR), and the C-terminal domain (CTD) [1]. To investigate the protein structure of GmARFs, SWISS-MODEL online software (https://swissmodel.expasy.org/, accessed on 23 November 2024) was used for protein structure predictions. The protein structures were categorized into repressor (Figure 4B) and activator (Figure 4C) types. Both repressor and activator protein structures share identical B3, Auxin-resp, and PB1 domains but exhibit significant differences in the MR domain. Specifically, the B3 domain consists of a β-barrel formed by seven parallel β-sheets and two α-helices (Figure 4B(b-I),C(b-I)). The Auxin-resp domain is composed of two α-helices at the N′-terminus connected to two β-strands at the C′-terminus (Figure 4B(b-II),C(b-II)). The PB1 domain is made up of five β-strands and two α-helices (Figure 4B(c-I),C(c-I)). The MR domain is divided into two distinct structural types. The repressor is mostly composed of two β-strands (Figure 4B(d-I)), with a high proportion of serine (Serine), leucine (Leucine), and proline (Proline) amino acids (Figure 4B-e). In contrast, the activator core proteins primarily consist of two α-helices (Figure 4C(d-I), with a higher proportion of serine (Serine), leucine (Leucine), and glutamine (Glutamine) amino acids (Figure 4C-e).

**Figure 5:**
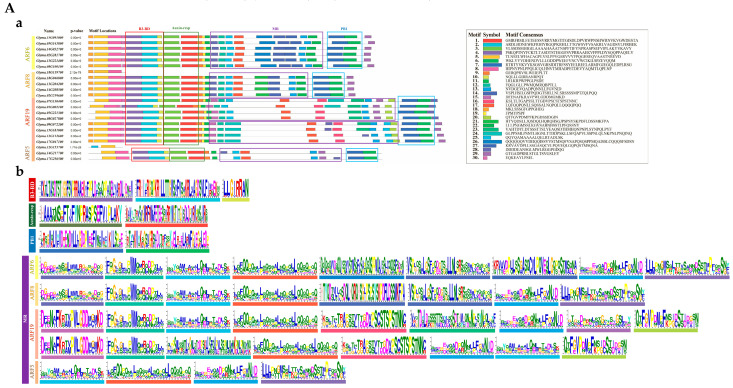

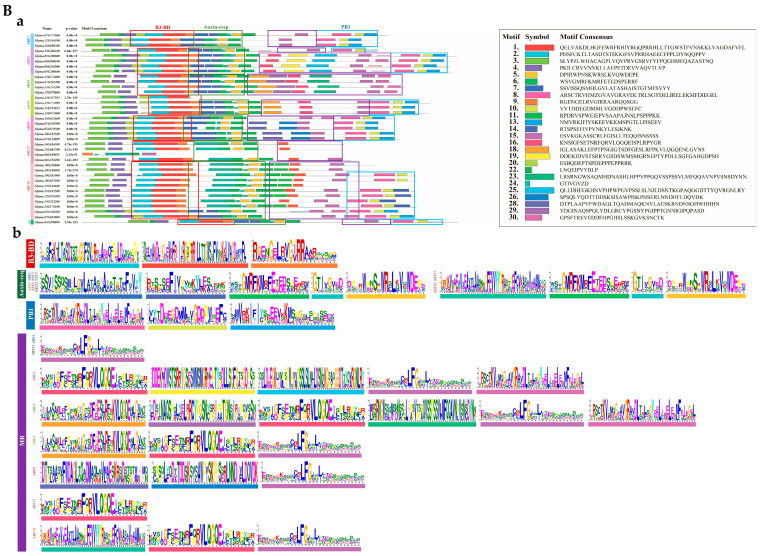
Analysis of amino acid motifs in the activator and repressor of GmARFs. (**A**) Structural diagram of activator GmARF motif; (**B**) structural diagram of repressor GmARF motif; (**A-a**) distribution map of activator GmARF motif. Each distinct colored block within a gene represents a unique motif. The red-bordered frame denotes the B3-BD domain, the green-bordered frame indicates the Auxin-responsive domain, the purple-bordered frame corresponds to the MR domain, and the blue-bordered frame marks the PB1 domain; (**B-a**) distribution map of repressor GmARF motif. Each distinct colored block within a gene represents a unique motif. The red-bordered frame denotes the B3-BD domain, the green-bordered frame indicates the Auxin-responsive domain, the purple-bordered frame corresponds to the MR domain, and the blue-bordered frame marks the PB1 domain; (**A-b**) amino acid information of activator GmARF motif. Each distinct colored block within a gene represents a unique motif; (**B-b**) amino acid information of repressor GmARFs. Each distinct colored block within a gene represents a unique motif.

To further explore the biological functions of the *GmARF* family genes, motif analysis was conducted on these members. The results showed that the motifs within the B3 domain, Auxin-resp domain, and PB1 domain were largely identical between repressor and activator members (Figure 5A-a,B-a). For activator subfamily members, the number of motifs in the MR domain ranged from 4 to 9, with a total of 14 distinct motifs identified. The small variation in motif types among subfamilies suggested high functional conservation and close evolutionary relatedness (Figure 5A-b). In contrast, repressor subfamily members exhibited greater diversity, with the motif number in the MR domain ranging from 1 to 6 and a total of 12 distinct motifs.

A comparison of the MR domain between subfamilies (Figure 5A-b,B-b) revealed that all activator members contained 13 and 17 motifs. In contrast, *GmARF19* exhibited two distinct types of motifs, indicating a higher degree of divergence within the family and potential functional differences. *GmARF5* had a relatively limited number of motif types in this structure. The repressor subfamily was relatively more complex. Most members contained 15 and 16 motifs. Among them, *GmARF1*, *GmARF17*, and *GmARF23* each had only one type of motif. However, *GmARF4*, *GmARF9*, and *GmARF18* each had three different types of motifs. Notably, *GmARF2* and *GmARF3* exhibited a high variety of motif types.

### 2.6. Cis-Regulatory Element Analysis of GmARF Genes

**Figure 6:**
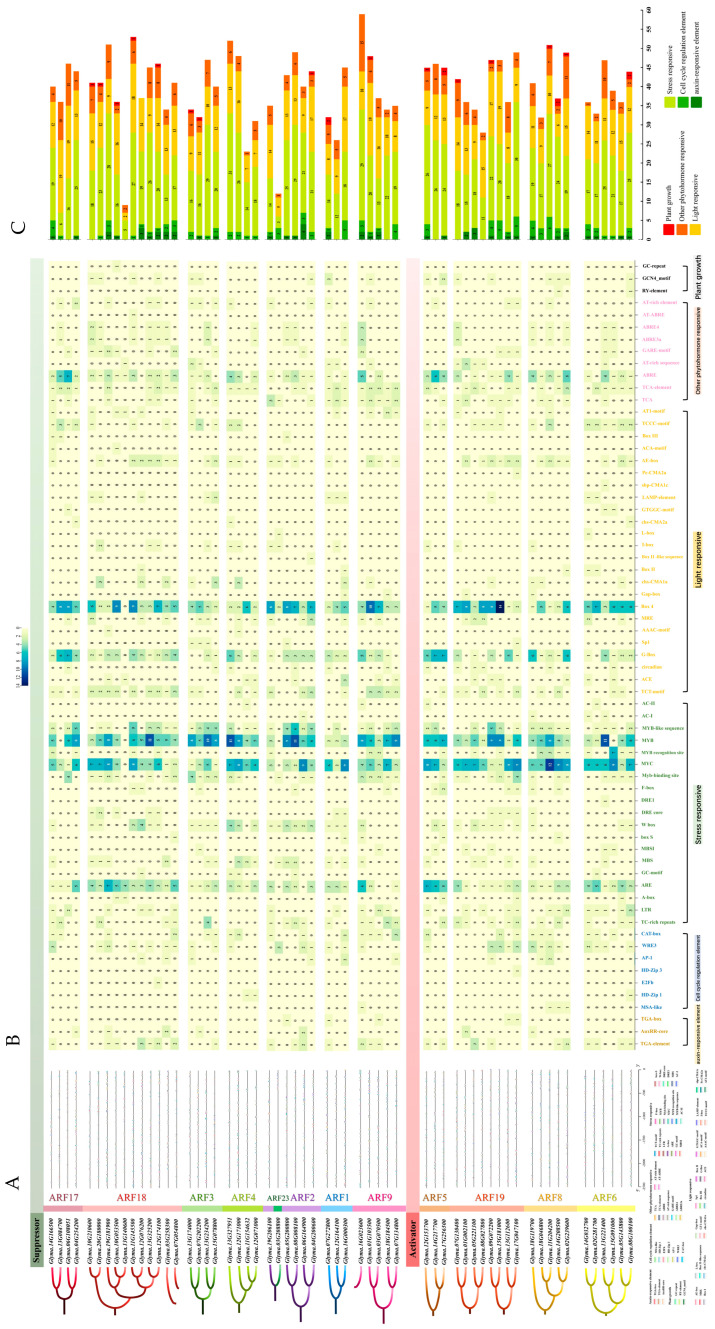
Analysis of cis-elements in the promoter of *GmARFs*. (**A**) Analysis of cis-elements in the promoter region of *GmARF* genes. (**B**) Heatmap of the number of cis-elements; the different colors represent the number of cis-elements in each category. (**C**) The sum of cis-elements in categories shown as a histogram.

To investigate the expression pattern and potential upstream regulators of *GmARF* transcription factors, the promoter regions with 2000bp upstream of the start codon of *GmARF* genes were analyzed and predicted using PlantCARE (Supplementary Table S1). A total of 65 cis-regulatory elements were identified and categorized into six groups: light-responsive elements (24, 36%), auxin-responsive elements (3, 5%), response elements for other plant hormones (9, 14%), stress-responsive elements (19, 29%), regulatory elements involved in plant growth and development (3, 5%), and regulatory elements for cell cycle changes (7, 11%) (Figure 6A). Among them, light-responsive elements accounted for a relatively high proportion, followed by response elements for other plant hormones and stress-responsive elements. Regulatory elements involved in plant growth and development, auxin-responsive elements, and regulatory elements for cell cycle changes were less abundant. This suggests that this gene family not only responds to changes in light but also might participate in multiple plant hormone regulatory mechanisms, reflecting the diversity of their functionalities.

Further in-depth analysis showed that MYB elements (317), MYC elements (302), Box4 elements (283), ARE elements (155), G-box elements (147), and ABRE elements (115) constitute a significant proportion within the promoters of this gene family (Figure 6B). Among them, MYB elements, MYC elements, and ARE elements are stress-responsive elements. Box4 elements and G-box elements are light-responsive elements. ABRE elements are responsive to other plant hormones. These findings indicate that this gene family might be involved in regulating abiotic stress mechanisms while also playing crucial roles in regulating photosynthesis, growth, and development and adapting to different light conditions. The auxin-responsive elements, including TCA-element (34), AuxRR-core (10), and TGA-box (5), account for a relatively small proportion, suggesting that most genes in this family might not be transcriptionally regulated by auxin directly.

Quantification analysis of cis-elements in the promoter *GmARF* gene family (Figure 6C) indicates that the number of stress-responsive elements (ranging from 5 to 31) is the highest, followed by the light-responsive elements (ranging from 7 to 19). There are fewer elements responsive to plant hormones (ranging from 1 to 15) and regulatory elements controlling cell cycle changes (ranging from 1 to 6). Regulatory elements controlling plant growth and development are the lowest (ranging from 1 to 4). These findings suggest that the *GmARF* genes could be directly regulated by various abiotic stresses and light and its participation in auxin signaling through interacting with other proteins.

Subfamily analysis revealed that activator subfamilies possess a higher number of cis-acting elements compared to repressors, with the quantity of various elements being more stable in activators than in repressors. Stress-responsive and light-responsive elements constitute a large proportion in the gene family sequences of repressors *GmARF2/3/4/9/18/23* and activators *GmARF5/6/8/19*, followed by stress-responsive elements and elements responsive to other plant hormones (Figure 6B). Among them, the number of auxin-responsive elements in the gene family sequences of repressors *GmARF2/9/18* and activators *GmARF6/8/19* ranges from 1 to 10, while the remaining subfamilies lack auxin-responsive elements. The repressor genes *GmARF18* (*Glyma.13G325200*), *GmARF3* (*Glyma.13G234200*), *GmARF4* (*Glyma.13G327951*), and *GmARF2* (*Glyma.08G008100*), as well as the activator gene *GmARF6* (*Glyma.13G221400*), have more MYB elements than other elements (Figure 6B). The repressor genes *GmARF2* (*Glyma.06G164900*) and *GmARF1* (*Glyma.16G000300*) and the activator genes *GmARF6* (*Glyma.15G091000*), *GmARF8* (*Glyma.11G204200*, *Glyma.14G208500*), and *GmARF19* (*Glyma.17G047100*) have more MYC elements than other elements. The repressor genes *GmARF18* (*Glyma.10G053500*, *Glyma.11G145500*) and *GmARF9* (*Glyma.01G103500*), as well as the activator gene *GmARF19* (*Glyma.15G181000*), have more Box4 elements than other elements in their promoter regions. The repressor genes *GmARF17* (*Glyma.13G084700*, *Glyma.06G108051*) and *GmARF9* (*Glyma.16G023600*), as well as the activator genes *GmARF5* (*Glyma.14G217700*) and *GmARF8* (*Glyma.02G239600*), have more ABRE elements than other elements in their promoters. This suggests that certain types of elements are clustered in specific genes, implying that these types of regulatory elements may be evolutionarily conserved.

### 2.7. Expression Analysis of GmARF Genes

**Figure 7:**
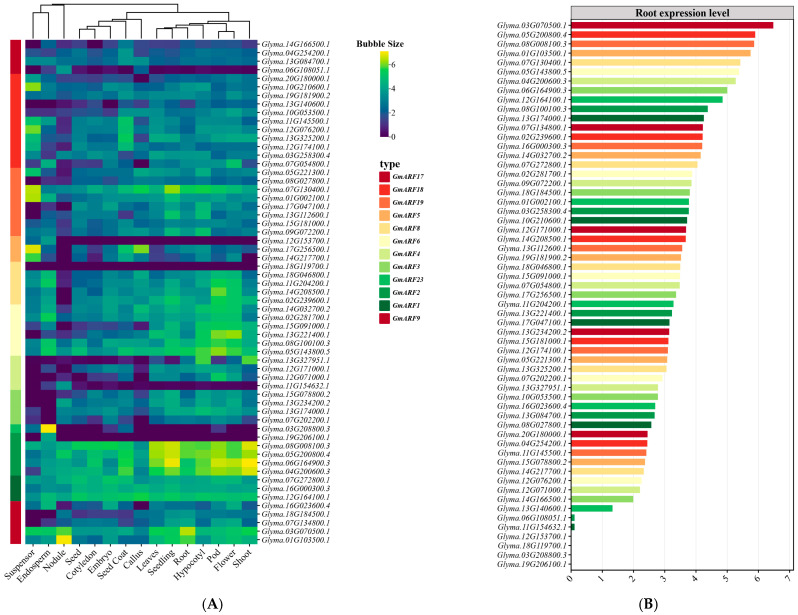
Expression analysis of *GmARF* families. (**A**) Heatmap depicting the expression patterns of *GmARFs* across various tissues. (**B**) Comparative analysis of *GmARF* expression levels in the roots of soybean.

An investigation into the tissue expression patterns of auxin response factor gene family members (*GmARFs*) in soybean (*Glycine max*) was conducted by utilizing the Phytozome database (http://www.phytozome.net/, accessed on 23 November 2024) and soybean expression atlas data (https://venanciogroup.uenf.br/cgi-bin/gmax_atlas/index.cgi, accessed on 23 November 2024). As illustrated in Figure 7A, results showed that most *GmARF* members are expressed in various tissues of the soybean plant. The activator subfamilies *GmARF6*, *GmARF8*, and *GmARF19* exhibited higher expression levels in aerial organs compared to underground organs, with the highest expression in flowers and pods, followed by hypocotyls. Notably, most subfamily members showed low expression in root nodules. Conversely, the repressor subfamilies *GmARF3*, *GmARF4*, *GmARF5*, *GmARF17*, and *GmARF18* showed higher expression in underground parts than in aerial organs. Among these, some members of the *GmARF3*, *GmARF4*, and *GmARF17* subfamilies were highly expressed in the nodules. The *GmARF1*, *GmARF2*, and *GmARF9* subfamilies exhibited high expression levels across all organs compared to the other subfamilies (Figure 7B). Specifically, the *GmARF1* and *GmARF2* subfamilies showed higher expression in aerial organs than in underground organs, with the highest expression in stems, flowers, and pods, followed by roots. In contrast, most members of the *GmARF9* subfamily were preferentially expressed in roots. Integrative analysis of root-specific expression profiling (Figure 7B) identified two genes, *Glyma.05G200800* (*GmARF2* subfamily) and *Glyma.03G070500* (*GmARF9* subfamily), with root-enriched expression patterns. Both genes exhibited 1–2-fold higher expression in roots compared to other organs and ranked among the top two most abundant of 59 *GmARF* genes in root tissues. Notably, *Glyma.05G200800* (*GmARF2*) and *Glyma.03G070500* (*GmARF9*) act as transcriptional repressors, whereas previous research has primarily focused on ARF activators, with scarce reports on ARF repressors. These two genes were thus prioritized as primary targets for further mechanistic studies.

### 2.8. Functional Study of GmARF9b (Glyma.03G070500) and GmARF2a (Glyma.05G200800) in Soybean

**Figure 8:**
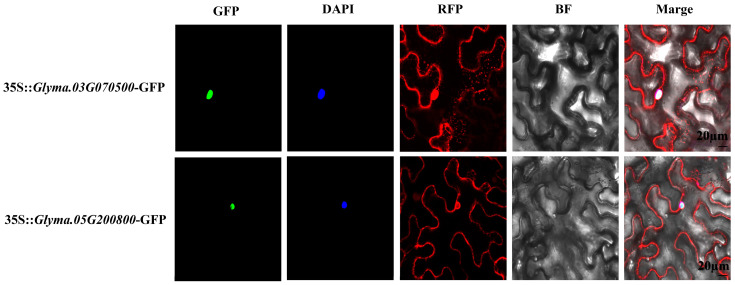
The subcellular localization of *GmARF9b*/*GmARF2a* in tobacco leaves. Red fluorescence corresponds to RFP (Red Fluorescent Protein) signals and RFP represents the RFP channel image. Blue fluorescence marks DAPI (4′,6-Diamidino-2-Phenylindole) nuclear staining and DAPI represents the DAPI channel image. Green fluorescence indicates GFP (Green Fluorescent Protein) signals and GFP represents the GFP channel image. BF (bright field) is the field-of-view image, and the scale bar is 20 µm.

A transient assay of tobacco leaves was used to determine the subcellular localization of GmARF9b (*Glyma.03G070500*) and GmARF2a (*Glyma.05G200800*) (Figure 8). As shown in the figure, *35S-GmARF9b*/*GmARF2a*-GFP was mainly localized in the nucleus, which was indicated by the DAPI staining. While the control *35S/RFP* was localized both in the nucleus and the cytoplasm. These results suggest that both *GmARF9b* and *GmARF2a* are mostly localized in the nucleus.

**Figure 9:**
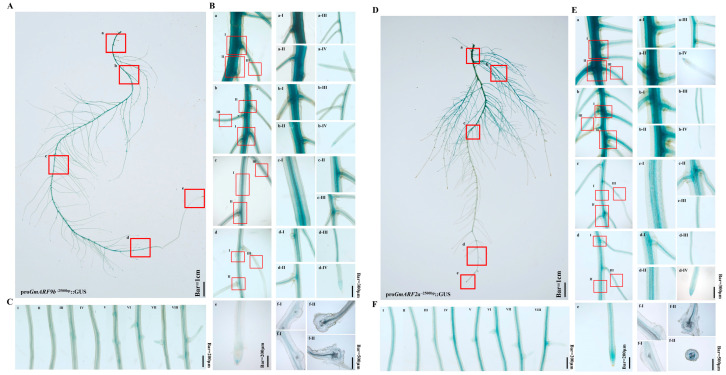
Tissue localization analysis of GmARF9b/GmARF2a in soybean hairy roots. (**A**) Tissue localization map of GmARF9b. The red-boxed areas in the figure correspond to subpanels a–e; (**B-a**) base of the primary root. The red-boxed areas in the figure correspond to subpanels **I**–**III**; (**B-a-I,II**) detailed view of the primary root base; (**B-a-III**) detailed view of first-order lateral root at the primary root base; (**B-a-IV**) detailed view of the first-order lateral root tip; (**B-b**) connection between the primary root and lateral roots. The red-boxed areas in the figure correspond to subpanels **I**–**III**; (**B-b-I,II**) detailed view of the primary root; (**B-b-III**) detailed view of the lateral root at the primary root; (**B-b-IV**) detailed view of the lateral root tip; (**B-c**) lateral root. The red-boxed areas in the figure correspond to subpanels **I–III**; (**B-c-I,II**) detailed view of the lateral root; (**B-c-III**) detailed view of second-order lateral root; (**B-d**) lateral root primordium. The red-boxed areas in the figure correspond to subpanels **I**–**III**; (**B-d-I,II**) detailed view of the lateral root primordium; (**B-d-III**) detailed view of second-order lateral root; (**B-d-IV**) detailed view of the lateral root tip; (**B-e**) tip of the primary root; (**B-f-I**) longitudinal sections of root cells from panel B-d; (**B-f-II**) transverse sections of root cells from panel B-d; (**C-I,II,III,IV,V,VI,VII,VIII**) formation process of *GmARF9b* lateral root primordium. (**D**) Tissue localization map of GmARF2a. The red-boxed areas in the figure correspond to subpanels a–e; (**E-a**) base of the primary root. The red-boxed areas in the figure correspond to subpanels **I**–**III**; (**E-a-I,II**) detailed view of the primary root base; (**E-a-III**) detailed view of first-order lateral root at the primary root base; (**E-a-IV**) detailed view of the first-order lateral root tip; (**E-b**) connection between the primary root and lateral roots. The red-boxed areas in the figure correspond to subpanels **I**–**III**; (**E-b-I,II**) detailed view of the primary root; (**E-b-III**) detailed view of the lateral root at the primary root; (**E-b-IV**) detailed view of the lateral root tip; (**E-c**) lateral root. The red-boxed areas in the figure correspond to subpanels **I–III**; (**E-c-I,II**) detailed view of the lateral root; (**E-c-III**) detailed view of second-order lateral root; (**E-d**) lateral root primordium. The red-boxed areas in the figure correspond to subpanels **I**–**III**; (**E-d-I,II**) detailed view of the lateral root primordium; (**E-d-III**) detailed view of second-order lateral root; (**E-d-IV**) detailed view of the lateral root tip; (**E-e**) tip of the primary root; (**E-f-I**) longitudinal sections of root cells from panel **E-d**; (**E-f-II**) transverse sections of root cells from panel **E-d**; (**F-I,II,III,IV,V,VI,VII,VIII**) formation process of *GmARF2a* lateral root primordium.

To determine the tissue expression pattern of *GmARF9b* and *GmARF2a*, promoters (with 2000 bp upstream of the ATG initiation codon) were cloned and fused upstream of the reporter gene GUS, respectively, and the results of transgenic hairy roots after GUS staining showed that *GmARF9b* and *GmARF2a* were predominantly expressed in the stele, root tips, and lateral root primordia (Figure 9B,E). This suggests that *GmARF9b* and *GmARF2a* might play crucial roles during root development stages and may be involved in regulating root growth and development (Figure 9C,F).

**Figure 10:**
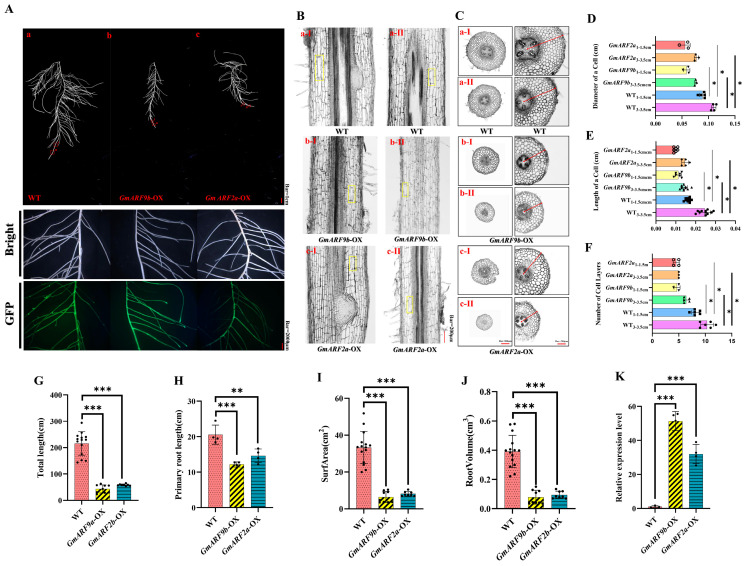
Effects of overexpression of *GmARF9b/GmARF2a* on root growth and development of soybean. (**A**) Phenotype images of *GmARF9b/GmARF2a* compared with control (CK) roots. (**A-a**) WT (*EV*); (**A-b**) *GmARF9b*-OX; (**A-c**) *GmARF2a*-OX. (**B**) Comparative longitudinal section views of cells. The yellow-boxed regions in the figure represent individual longitudinally sectioned cells. (**B-a-I**) Longitudinal sections of cells were obtained from the WT material at a distance of 1.0–1.5 cm from the root apex; (**B-a-II**) longitudinal sections of cells were obtained from the WT material at a distance of 3.0–3.5 cm from the root apex; (**B-b-I**) longitudinal sections of cells were prepared from the *GmARF9b*-OX material at a distance of 1.0–1.5 cm from the root apex; (**B-b-II**) longitudinal sections of cells were prepared from the *GmARF9b*-OX material at a distance of 3.0–3.5 cm from the root apex; (**B-c-I**) longitudinal sections of cells were prepared from the *GmARF2a*-OX material at a distance of 1.0–1.5 cm from the root apex; (**B-c-II**) longitudinal sections of cells were prepared from the *GmARF2a*-OX material at a distance of 3.0–3.5 cm from the root apex. (**C**) Comparative transverse section views of cells; (**C-a-I**) transverse sections of cells were obtained from the WT material at a distance of 1.0–1.5 cm from the root apex; (**C-a-II**) transverse sections of cells were obtained from the WT material at a distance of 3.0–3.5 cm from the root apex; (**C-b-I**) transverse sections of cells were prepared from the *GmARF9b*-OX material at a distance of 1.0–1.5 cm from the root apex; (**C-b-II**) transverse sections of cells were prepared from the *GmARF9b*-OX material at a distance of 3.0–3.5 cm from the root apex; (**C-c-I**) transverse sections of cells were prepared from the *GmARF2a*-OX material at a distance of 1.0–1.5 cm from the root apex; (**C-c-II**) transverse sections of cells were prepared from the *GmARF2a*-OX material at a distance of 3.0–3.5 cm from the root apex. (**D**) Cell diameter. (**E**) Cell length. (**F**) Number of cell layers. (**G**) Total root length. (**H**) Root volume. (**I**) Root surface area. (**J**) Primary root length. (**K**) Comparison of GFP expression levels. The asterisks “*”, “**”, and “***” indicate significance at the 5% (*p* < 0.05) level, while “ns” indicates no significant difference.

To further investigate the roles of *GmARF9b* and *GmARF2a* in regulating soybean root growth and development, overexpression of *GmARF9b* and *GmARF2a* driven by 35S was constructed and transformed into the hairy roots of soybean. Positive transgenic hairy roots were identified by a GFP marker using fluorescence microscope at the GFP channel (Figure 10A). Results showed that the hairy roots overexpressing *GmARF9b* and *GmARF2a* exhibited significant decreases in total root length by 72.84–79.92%, decreases in primary root length by 40.63–69.05%, 75.21–80.53% decreases in root surface area, and 75.67–79.82% decreases in root volume compared with wild-type hairy roots (Figure 10G–J). Furthermore, the root section in the 3–3.5 cm of root segment showed that the cell diameter, cell length, and cell layer number were significantly lower in the OE transgenic hairy roots by 42.24–44.73%, 68.12–71.97%, and 61.46–106.67% compared with the control hairy roots (Figure 10D–F). Similar results were also observed in the 1–1.5 cm root segment with a cell diameter, cell length, and cell layer number significantly reduced by 50.51–58.57%, 43.99–64.86%, and 75–81.48%, respectively. Furthermore, the natural variations in *GmARF9b* and *GmARF2a* were analyzed, and the haplotypes of both genes under selection were identified (Supplementary Figures S1 and S2; Supplementary Table S2). Taken together, these findings suggest that *GmARF9b* and *GmARF2a* negatively regulate soybean root growth and development, with impacts on root cell division and elongation.

## 3. Discussion

### 3.1. Phylogenetic Analysis, Classification, and Gene Haplotype of GmARF Genes

ARF is a key factor in auxin signaling transduction in plants, and this family plays a crucial role in plant growth and development. Initially, 51 members of the soybean ARF gene family were identified [18]. In this study, we conducted BLAST (ElasticBLAST 1.4.0) analysis in the new version of the soybean genome [19]. Our results suggest that there are 59 ARF gene members in the soybean genome (Figure 1A). Previous studies classified GmARF family members into five subclasses and three major branches [18]. In this study, using the updated GmARF protein members and amino acid sequences, our results suggested that GmARF family members could be divided into four subclasses and two major branches. Our results showed two branches were divided according to their potential functions, one branch belonging to the repressor subfamily and the other belonging to the activator subfamily (Figure 1A). Therefore, our phylogenetic tree analysis was more corelative with the potential protein functions. On the other hand, among the 59 soybean members, subfamilies *ARF10*, *ARF16*, *ARF7*, *ARF11*, *ARF12*, *ARF13*, *ARF14*, *ARF15*, *ARF20*, *ARF21*, and *ARF22* in *Arabidopsis thaliana* are absent in soybean (Figure 1A) [20]. This phenomenon may be attributed to a significant subfamily preference in the distribution of the *GmARF* gene family, where soybeans in subclass II may be more inclined to retain or evolve specific subfamily genes. The missing subfamilies may have been lost due to natural selection, genetic drift, or other evolutionary events during soybean evolution [21].

The gene distribution pattern across different chromosomes reveals that 13.72% of *GmARF* family members are clustered on chromosome 13 (chr13). This finding is consistent with the results of a previous study [18]. In addition, two new identified ARFs located in chromosome 12 were found through comparison with a previous study (Figure 1C). This discrepancy may be attributed to the more complete genome assembly of gene family members, leading to an increase in the number of genes on different chromosomes [17]. Such duplications are common evolutionary mechanisms that can expand gene families and potentially contribute to functional diversification within the family.

### 3.2. Dynamic Evolution of GmARF Gene Haplotypes Under Natural Selection in Soybean

Natural selection analysis is a crucial approach to understanding how genes are selected during evolution and how they influence plant phenotypes and adaptability. Previous studies have performed natural selection analysis on *GmARF* members [21]. In this study, we used the soybean database to perform haplotype analysis on the *GmARF* family [17]. Our findings revealed that no haplotype was observed in the repressor GmARF4/23 subfamily members. This suggests that these family members may be under strong selective pressure, resulting in a low rate of genetic variation [22]. Additionally, among the repressor *GmARF17* members, only *Glyma.14G166500* was subject to natural selection, while other members either lacked different haplotypes or were less impacted by natural selection. This could be due to the specific gene function and genetic variation in *Glyma.14G166500*. In contrast, other members may have redundant functions or genetic variations that are not conducive to the evolutionary environment [17].

### 3.3. Divergence of Conserved Motifs in GmARF Protein Domains

To facilitate subsequent research on gene function, comparative classification, and protein interaction mechanisms, this study analyzed gene protein structures and motifs based on functional classification (Figure 3). The results indicate that the repressor Auxin-resp domain is divided into two categories of motifs, and the motifs within the MR (middle region) domain exhibit relatively greater length and diversity compared to activators [20]. This significant difference may arise from the distinct selective pressures faced by gene members during evolution, leading to higher motif diversity in the repressor MR domain and relative uniformity in the activator MR domain [20]. Additionally, this suggests that repressors may recognize and bind to a variety of different proteins or DNA sequences, implying a more complex genetic molecular network.

### 3.4. Biological Function Analysis of GmARF9b (Glyma.03G070500) and GmARF2a (Glyma.05G200800) in Glycine Max

Based on expression level analysis, the genes *GmARF9b* (*Glyma.03G070500*) and *GmARF2a* (*Glyma.05G200800*) were identified as highly expressed in roots. To further elucidate the potential functions of these two genes, this study employed transgenic hairy roots for the functional study of *GmARF9b* (*Glyma.03G070500*) and *GmARF2a* (*Glyma.05G200800*). Transgenic hairy roots overexpressing *GmARF9b* and *GmARF2a* significantly inhibit root growth and development, with reduced root fresh weight, total root length, root volume, and surface area in OE lines (Figure 8). This demonstrates that *GmARF9b* (*Glyma.03G070500*) and *GmARF2a* (*Glyma.05G200800*) negatively regulate root growth and development. Subsequent cellular comparisons of the OE (*GmARF9b*-OE and *GmARF2a*-OE) transgenic hairy roots with *WT* (*EV*) hairy roots revealed that the overall cell area and length in the overexpression lines were significantly lower than those in the *WT* (Figure 10D,E), indicating that these genes negatively affect the cell elongation and the cell division and proliferation, thus repressing root growth and development. Given the relatively high proportion of cell cycle-related cis-acting elements in the promoters of these two *GmARF* genes (Figure 6B,C), further experiments are needed to uncover the underlying molecular mechanisms.

On the other hand, *GmARF* as a pivotal transcription factor in auxin signal transduction influences root growth and development through the regulation of downstream genes. Notably, *GmARF9b* and *GmARF2a* are identified as repressors within the ARF gene family, yet their molecular mechanisms remain unreported. However, based on previous studies, when auxin levels rise, Aux/IAA proteins undergo degradation, releasing ARFs. The accumulation of repressor-type ARFs is subsequently enhanced, leading to strengthened repression of downstream LBD genes. This results in reduced LBD protein accumulation, thereby inhibiting the elongation and development of both primary and lateral roots [23]. Given that auxin accumulates in the root tip, the increased IAA content therein activates the transcription of repressor-type ARFs. These ARFs bind to the promoters of downstream genes such as RSL to modulate their expression. The RSL protein, in turn, directly transcriptionally activates LRH, forming an LRH-RSL complex that suppresses root hair growth [24].

Additionally, previous studies on ARFs have primarily focused on model plants, particularly activators. For example, both forward and reverse genetic studies in *Arabidopsis thaliana* have confirmed that the activators *AtARF7* and *AtARF19* are key genes regulating lateral root formation, with phenotypic assays demonstrating functional redundancy between these two genes [14,15]. Furthermore, the activator *AtARF5* interacts with the promoters of *AtPIN1*, *AtPIN3*, and *AtPIN7* to regulate root meristem development, lateral root primordium formation, and lateral organ formation in the shoot apical meristem [10,11]. Therefore, the identification in this study of genes from soybean repressors that regulate root growth and development lays a theoretical foundation for future research on *GmARF* genes.

Taken together, in this study, we conducted a comprehensive investigation of the ARF gene family in soybean and functionally characterized ARFs that are highly expressed in roots. We identified two ARFs that negatively regulate root growth and development through modulating the cell elongation and cell division and proliferation in the roots. This study unveils the genetic mechanisms of the ARF gene family in the growth and development of soybean roots, providing new insights into deciphering the complex regulatory network of plant root development. However, the functions of *GmARF9b* and *GmARF2a* have not been linked to their natural variations, which still need further elite natural variations identification. Furthermore, these findings offer theoretical support and candidate genes for cultivating new soybean lines through molecular breeding approaches.

## 4. Materials and Methods

### 4.1. Phylogenetic Tree Analysis of the ARF Gene Family

Protein sequences of 23 AtARFs from *Arabidopsis thaliana* were downloaded from the TAIR10 database (http://www.arabidopsis.org accessed on 23 November 2024) [25]. Additionally, protein sequences of 59 GmARFs from *Glycine max* were obtained from the Phytozome13 database (https://phytozome-next.jgi.doe.gov/ accessed on 23 November 2024) [26]. The MUSCLE algorithm in the align function of MEGA5.2 software [27] was utilized to align the amino acid sequences of ARFs. The results of alignments were then subjected to phylogenetic analysis using the neighbor-joining (NJ) method in MEGA5.2 software, with the bootstrap method selected for branch length testing and a bootstrap value set at 1000 to generate the phylogenetic tree. Further visualization of the phylogenetic tree was carried out using the online tool iTOL (https://itol.embl.de/login.cgi?logout=1 accessed on 26 November 2024) [28].

### 4.2. Chromosome Distribution and Gene Duplication Analysis of ARFs

To investigate the chromosomal locations of ARFs, positional information for ARFs was extracted from the genomic annotation files of soybean (Glycine max) and Arabidopsis thaliana. Circos (circos-0.69-9) software was employed to map the positions of GmARFs onto their respective chromosomes [29]. To analyze gene duplication events, the Multicollinearity Scanning Toolkit (MCScanX) was utilized with default parameters [30]. The results obtained from MCScanX were visualized using the TBtools (TBtools-ll v2.210) [31]. To construct the synteny between ARFs of soybean and Arabidopsis, the Graphics function in TBtools was leveraged for dual-genome synteny analysis [31]. This analysis ultimately yielded the syntenic relationships between the ARFs of the two species.

### 4.3. Haplotype Analysis of GmARF Gene Family

Haplotype analysis was conducted using SoyFGBv2.0 (https://sfgb.rmbreeding.cn/analysis/haplotype accessed on 26 November 2024) [31]. This database encompasses 2214 soybean core germplasm resources from four major soybean production and distribution regions: Asia, America, Europe, and Africa. Among these, there are 1993 cultivated varieties, 218 annual wild species, and 2 perennial wild species (*G. tomentella*) along with one *G. tabacina* [32].

### 4.4. Gene Structure and Protein Conserved Domain Prediction of GmARFs

Protein and gene sequences for 59 GmARFs from *Glycine max* were downloaded from the Phytozome database (https://phytozome-next.jgi.doe.gov/ accessed on 23 November 2024) [33]. TBtools was utilized to read the whole-genome annotation files, allowing the extraction of positions and sequences for exons, introns, promoters, terminators, and other elements. With this information, the Visualize Gene Structure function in TBtools was employed to further analyze and visualize the gene structures and features of the GmARF gene family. Additionally, the GXF Sequences Extract plugin in TBtools was used to predict conserved domains within the protein sequences of the GmARFs, and the results were visualized accordingly.

### 4.5. Whole-Genome Characterization Analysis of the GmARF Gene Family in Soybean

Based on the amino acid sequences of ARFs, the whole-genome characteristics data for the *GmARF* genes were collected using the BLAST function available at NCBI (https://blast.ncbi.nlm.nih.gov/Blast.cgi accessed on 20 November 2024) [34]. Additionally, subcellular localization predictions for the genes in the *GmARF* family were performed using WoLF PSORT (https://www.genscript.com/tools/wolf-psort accessed on 26 November 2024) [35].

### 4.6. Protein Structure Prediction and Motif Analysis of the GmARFs Gene Family in Soybean

The InterPro database (https://www.ebi.ac.uk/interpro/ accessed on 23 November 2024) was used to integrate protein domains and functional sites of the *GmARF* gene family [33]. Based on this information, the conserved protein domains were visualized. Additionally, SWISS-MODEL (https://swissmodel.expasy.org/interactive accessed on 23 November 2024) was employed to predict the protein structures by aligning amino acid sequences with similar known structures, and the quality of the predictions was evaluated using metrics such as GMQE, QMEAND, and Co global [36]. The final output was a visualized 3D structure, accompanied by data integration based on the proportion of amino acids.

Furthermore, the online software MEME (http://meme-suite.org/tools/meme accessed on 27 November 2024) was utilized by inputting the amino acid sequences of the proteins [37]. The number of motifs prediction was set to 30, with lengths ranging from 10 to 50 amino acids. The resulting motif sequences were integrated based on information such as the position and score of each motif.

### 4.7. Cis-Regulatory Element Analysis of the GmARF Gene Family in Soybean

The nucleic acid sequences 2000 bp upstream of the start codon for each member of the GmARF gene family were extracted and analyzed using TBtools. These sequences were then analyzed for cis-regulatory elements within the promoter regions using the PlantCARE online tool (https://bioinformatics.psb.ugent.be/webtools/plantcare/html/ accessed on 27 November 2024) [38]. The results of prediction were visualized using the Simple BioSequence Viewer (TBtools-ll v2.210) in TBtools. Based on the functional classification of the cis-regulatory elements, the number and proportion of each element were calculated and integrated.

### 4.8. Analysis of Gene Expression Patterns of the GmARF Family in Soybean

The expression pattern of GmARF gene family across different soybean organs were analyzed using the Soybean Expression Atlas (https://venanciogroup.uenf.br/cgi-bin/gmax_atlas/index.cgi accessed on 23 November 2024) online software [39].

### 4.9. Acquisition of Transgenic Hairy Roots Overexpressing GmARF9b (Glyma.03G070500) and GmARF2a (Glyma.05G200800) in Soybean

Primers were designed based on the ORF sequences of *GmARF9b* (*Glyma.03G070500*) and *GmARF2a* (*Glyma.05G200800*). Using KOD One (Vazyme, Nanjing, China) high-fidelity enzyme, fragments were amplified separately from a cDNA library (Supplementary Table S3). The fragments were then cloned into the pFGC5941-35S-intron-(GFP-Bar marker) overexpression vector using the Clon Express II One Step Cloning Kit with homologous recombination enzyme (Vazyme, Nanjing, China), utilizing the restriction sites *Asc* I (New England Biolabs, Ipswich, MA, USA) and *Bam*H I (New England Biolabs, Ipswich, MA, USA). The constructs were transformed into Agrobacterium rhizogenes K599 (Weidi Biotechnology, Shanghai, China).

The specific procedure was as follows [40]: Soybean seedlings were germinated for 6 days and then removed from vermiculite and washed. A diagonal cut was made 1–1.5 cm below the hypocotyl, and an appropriate amount of Agrobacterium cells was applied to the cut surface. The seedlings were then carefully placed on sterile water-soaked filter paper with the cut surface facing up, covered with plastic wrap to maintain moisture, and cultured in the dark for 4–5 days. Once the cut surface swelled and callus was induced, the seedlings were transferred to vermiculite and continued to be cultured for about a week. When the hairy roots grew to 1–2 cm, positive roots were screened by fluorescence observation and could be transferred to corresponding full-nutrient hydroponic cultures for further treatment.

### 4.10. Tissue Localization Analysis of GmARF9b (Glyma.03G070500) and GmARF2a (Glyma.05G200800) in Soybean

Soybean hairy roots were collected and placed in 50 mL centrifuge tubes. GUS staining solution was added to the tubes, composed of 50 mM sodium phosphate buffer (pH 7.0), 0.1% (*v*/*v*) Triton X-100 (Sigma-Aldrich, St. Louis, MO, USA), 0.1 mM K_3_Fe(CN)_6_, 0.1 mM K_4_[Fe(CN)_6_]•3H_2_O, 1 mg/mL X-Gluc, and 1% (*v*/*v*) dimethylformamide [41]. The samples were completely submerged in the GUS staining solution and incubated at 37 °C for 12 h. Subsequently, the samples were eluted with 95% ethanol and observed under an optical microscope (Axio Zoom; Zeiss, Oberkochen, Germany) to visualize the GUS-stained tissues.

### 4.11. Subcellular Localization of GmARF9b (Glyma.03G070500) and GmARF2a (Glyma.05G200800) in Soybean

Specific primers containing restriction enzyme sites were designed based on the ORF sequences of *GmARF9b* (*Glyma.03G070500*) and *GmARF2a* (*Glyma.05G200800*). The CDS regions of *GmARF9b* and *GmARF2a* were amplified using their respective ORF sequences as templates, and *Asc* I (New England Biolabs, Ipswich, MA, USA) enzyme digestion was performed (Supplementary Table S3). These fragments were then cloned into the *p5941-35S-GmARF9b/GmARF2a-GFP* subcellular localization expression vectors. The plasmids with correct sequencing results were transformed into *Agrobacterium* EHA105 (Weidi Biotechnology, Shanghai, China). After PCR detection, positive strains were selected and activated at 28 °C with shaking at 250 rpm for 2 days in a 1:1000 ratio. The cultures were centrifuged at 7000× *g* rpm for 8 min, and the supernatant was discarded. The OD_600_ of the resuspended cells in infiltration buffer (containing 10 mmol/L MgCl_2_, 10 mmol/L MES, and 200 µmol/L AS) was adjusted to 0.8–1.2 for use as the infiltration suspension. The infiltration of tobacco leaves was performed according to a previously described study [42]. After co-culturing for two days, the tobacco leaves were observed using a laser scanning confocal microscope for imaging.

## Figures and Tables

**Table 1:** Collection of gene sequences and protein information for members of the soybean family.

Classification	Gene Name	Gene Loci	Chromosome	Gene Position		Size (aa)	MW (Da)	PI	Instability Index	A.I.	GRAVY	Predicted Location	Arabidopsis Orthologues	
				Start	End								Gene Name	Gene Loci
Suppressor	*GmARF1*	*Glyma.07G272800*	chr07	44,814,499	44,821,841	701	78,043.97	5.83	58.93	75.48	−0.475	Nucleus	*AtARF1*	*AtlG59750*
		*Glyma.12G164100*	chr12	33,328,006	33,334,622	665	74,273.52	5.75	60.82	67.98	−0.523	Nucleus		
		*Glyma.16G000300*	chr16	25,166	31,978	666	74,250.40	5.62	58.72	70.36	−0.494	Nucleus		
	*GmARF2*	*Glyma.05G200800*	chr05	38,470,758	38,476,414	858	94,845.13	6.35	52.13	67.02	−0.638	Nucleus	*AtARF2*	*At5G62000*
		*Glyma.08G008100*	chr08	643,657	649,224	853	95,522.61	6.50	53.98	71.98	−0.577	Nucleus		
		*Glyma.06G164900*	chr06	13,626,690	13,632,154	843	93,667.20	6.19	59.40	63.49	−0.654	Nucleus		
		*Glyma.04G200600*	chr04	46,137,859	46,143,355	850	94,721.43	6.35	56.01	64.93	−0.661	Nucleus		
		*Glyma.19G206100*	chr19	46,694,888	46,706,151	677	75,645.75	7.11	57.20	73.87	−0.489	Nucleus		
	*GmARF3*	*Glyma.13G174000*	chr13	28,226,449	28,232,503	714	78,286.47	6.67	54.87	70.31	−0.400	Nucleus	*AtARF3*	*At2G33860*
		*Glyma.07G202200*	chr07	37,447,174	37,452,876	709	77,934.52	6.67	52.83	73.27	−0.358	Nucleus		
		*Glyma.13G234200*	chr13	33,897,474	33,903,871	739	80,750.89	6.18	55.82	75.20	−0.315	Nucleus		
		*Glyma.15G078800*	chr15	6,035,900	6,041,756	736	80,922.02	6.10	54.51	75.50	−0.309	Plasmodesmata		
	*GmARF4*	*Glyma.13G327951*	chr13	41,634,392	41,640,306	254	28,301.65	5.23	54.88	66.81	−0.498	Nucleus	*AtARF4*	*At5G60450*
		*Glyma.12G171000*	chr12	34,108,206	34,114,864	799	88,732.31	6.49	55.71	70.53	−0.438	Nucleus		
		*Glyma.11G154632*	chr11	11,580,788	11,587,787	792	88,149.47	5.98	53.35	74.91	−0.433	Nucleus		
		*Glyma.12G071000*	chr12	5,182,536	5,190,631	792	87,731.72	6.00	53.18	73.83	−0.428	Nucleus		
	*GmARF9*	*Glyma.18G184500*	chr18	44,742,372	44,749,324	664	74,553.01	5.89	51.79	76.46	−0.472	Nucleus	*AtARF9*	*At4G23980*
		*Glyma.07G134800*	chr07	15,844,232	15,849,865	664	74,516.94	5.99	51.99	74.23	−0.501	Nucleus		
		*Glyma.03G070500*	chr03	16,448,588	16,454,400	691	76,860.43	6.02	46.87	68.67	−0.537	Nucleus		
		*Glyma.01G103500*	chr01	35,187,770	35,192,407	692	76961.62	6.02	46.67	70.36	−0.504	Nucleus		
		*Glyma.16G023600*	chr16	2,242,818	2,248,084	717	79,581.75	6.14	53.34	72.73	−0.474	Nucleus		
	*GmARF17*	*Glyma.14G166500*	chr14	42,041,638	42,046,150	548	60,511.91	6.36	53.03	63.70	−0.389	Chloroplast	*AtARF17*	*At1G77850*
		*Glyma.13G084700*	chr13	18,614,254	18,618,647	551	60,677.92	5.78	52.46	63.36	−0.386	Chloroplast		
		*Glyma.06G108051*	chr06	8,647,276	8,649,645	162	18,067.40	6.85	51.29	71.48	−0.478	Cytoplasm		
		*Glyma.04G254200*	chr04	50,908,674	50,913,759	562	61,209.95	5.53	48.05	71.46	−0.265	Chloroplast		
	*GmARF18*	*Glyma.19G181900*	chr19	44,488,211	44,492,387	700	77,788.51	8.30	50.38	71.20	−0.378	Nucleus	*AtARF18*	*At3G61830*
		*Glyma.13G140600*	chr13	24,398,045	24,400,077	514	57,052.25	8.93	45.45	74.94	−0.344	Nucleus		
		*Glyma.10G053500*	chr10	4,804,493	4,809,528	700	77,266.44	7.26	46.42	73.39	−0.389	Nucleus		
		*Glyma.11G145500*	chr11	17,400,515	17,404,144	697	76,555.96	7.85	43.90	74.42	−0.324	Nucleus		
		*Glyma.12G076200*	chr12	5,836,558	5,840,461	701	77,188.67	8.24	43.03	73.59	−0.348	Nucleus		
		*Glyma.13G325200*	chr13	41,405,939	41,408,913	670	73,760.45	7.61	48.92	75.99	−0.331	Nucleus		
		*Glyma.12G174100*	chr12	34,605,359	34,608,860	700	76,824.93	7.60	48.61	75.07	−0.361	Nucleus		
		*Glyma.10G210600*	chr10	44,358,942	44,362,451	612	67,127.69	7.21	45.48	67.70	−0.397	Nucleus		
		*Glyma.20G180000*	chr20	41,744,451	41,747,715	593	65,246.96	6.70	46.04	70.20	−0.336	Nucleus		
		*Glyma.03G258300*	chr03	46,452,008	46,456,771	669	74,921.72	6.65	59.84	69.46	−0.516	Nucleus		
		*Glyma.07G054800*	chr07	4,782,625	4,787,849	716	79,530.35	6.07	52.65	70.80	−0.528	Nucleus		
	*GmARF23*	*Glyma.03G208800*	chr03	42,723,037	42,743,487	786	88,027.81	8.38	51.78	86.07	−0.235	Plasmodesmata	*AtARF23*	*AtlG43950*
Activator	*GmARF6*	*Glyma.14G032700*	chr14	2,368,563	2,377,330	898	99,065.45	6.13	66.79	75.04	−0.414	Nucleus	*AtARF6*	*At1G30330*
		*Glyma.02G281700*	chr02	48,225,437	48,235,389	896	98,952.51	6.04	67.86	75.31	−0.410	Nucleus		
		*Glyma.13G221400*	chr13	32,864,280	32,871,326	896	99,837.22	6.28	60.45	71.91	−0.497	Nucleus		
		*Glyma.15G091000*	chr15	6,970,378	6,977,273	898	99,689.89	6.22	57.02	73.82	−0.476	Nucleus		
		*Glyma.05G143800*	chr05	33,795,530	33,802,884	909	100,674.79	6.13	66.68	70.87	−0.483	Nucleus		
		*Glyma.08G100100*	chr08	7,676,663	7,683,563	907	100,662.05	6.17	64.72	72.21	−0.483	Nucleus		
	*GmARF8*	*Glyma.18G119700*	chr18	14,818,239	14,819,381	119	13,215.88	4.99	59.26	82.69	−0.364	Cytoplasm	*AtARF8*	*At5G37020*
		*Glyma.18G046800*	chr18	4,068,582	4,076,135	841	93,247.82	5.93	57.98	76.05	−0.441	Nucleus		
		*Glyma.11G204200*	chr11	3,3637,709	33,645,524	844	93,600.09	5.93	58.73	75.89	−0.461	Nucleus		
		*Glyma.14G208500*	chr14	48,263,889	48,272,154	843	93,795.44	6.01	60.63	74.12	−0.473	Nucleus		
		*Glyma.02G239600*	chr02	44,642,958	44,654,516	847	94,202.81	6.06	61.67	73.55	−0.495	Nucleus		
	*GmARF19*	*Glyma.07G130400*	chr07	15,309,926	15,315,946	1110	122,978.03	6.30	63.79	75.15	−0.578	Nucleus	*AtARF19*	*At1G19220*
		*Glyma.01G002100*	chr01	294,373	300,422	1104	121,957.83	6.51	63.38	76.10	−0.561	Nucleus		
		*Glyma.05G221300*	chr05	40,123,000	40,129,871	1099	120,828.59	6.01	61.52	80.35	−0.455	Nucleus		
		*Glyma.08G027800*	chr08	2,218,615	2,225,900	1113	122,551.22	5.86	63.04	77.85	−0.503	Nucleus		
		*Glyma.09G072200*	chr09	7,436,392	7,445,095	1125	125,840.87	6.02	67.64	67.01	−0.698	Nucleus		
		*Glyma.15G181000*	chr15	17,521,997	17,530,209	1122	125,284.27	6.19	67.09	67.9	−0.686	Nucleus		
		*Glyma.13G112600*	chr13	21,613,425	21,621,047	1131	126,747.12	6.08	65.28	73.47	−0.673	Nucleus		
		*Glyma.17G047100*	chr17	3,563,891	3,571,811	1136	127,058.28	6.05	67.23	72.90	−0.683	Nucleus		
	*GmARF5*	*Glyma.12G153700*	chr12	23,224,445	23,225,261	109	120,50.98	7.86	46.01	83.21	−0.184	Cytoplasm	*AtARF5*	*At1G19850*
		*Glyma.14G217700*	chr14	49,093,824	49,100,973	930	103,499.15	5.62	53.23	78.17	−0.407	Nucleus		
		*Glyma.17G256500*	chr17	41,134,622	41,140,694	933	103,492.16	5.48	51.50	77.19	−0.384	Nucleus		

## Data Availability

This study is an original research paper. All data used in this study are included in the main text and Supplementary Material of this paper. For any further questions, please contact the corresponding author of this article.

## Supplementary Materials

The following supporting information can be downloaded at: https://www.mdpi.com/article/10.3390/ijms26104547/s1.
