# Supplementary material for: Genome-Wide Identification of GmARF9b/GmARF2a Negatively Regulate Root Growth in Soybean
Source: Int J Mol Sci. 2025 May 9;26(10):4547. doi: 10.3390/ijms26104547 (PMC12110875; doi:10.3390/ijms26104547)
Supplement: Supplementary file 1 [file ijms-26-04547-s001.zip › Supplementary Figure.pptx]

## Slide 1
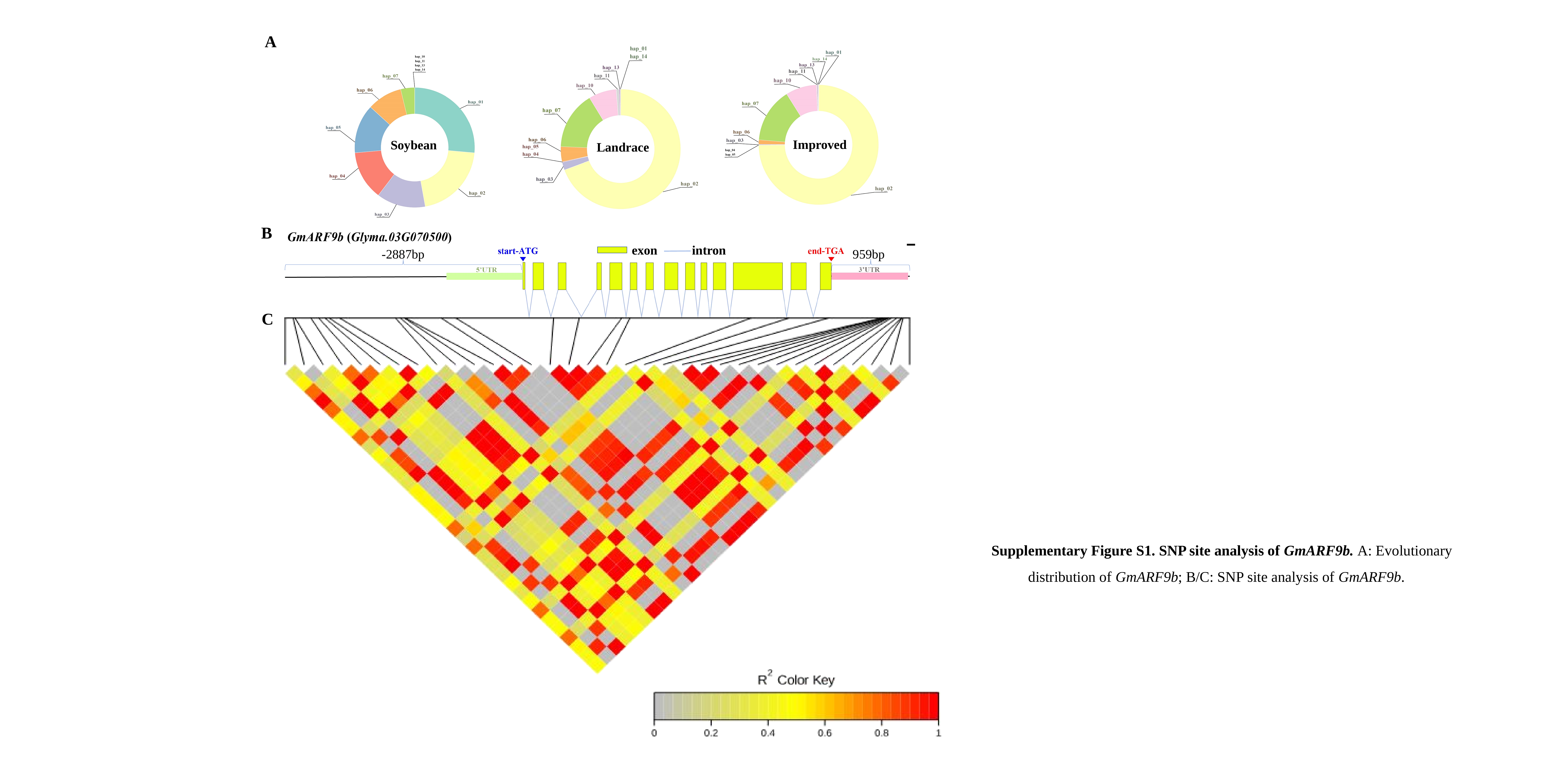

Improved
Soybean
Landrace
exon
intron
959bp
-2887bp
A
B
C
Supplementary Figure S1. SNP site analysis of GmARF9b. A: Evolutionary distribution of GmARF9b; B/C: SNP site analysis of GmARF9b.

## Slide 2
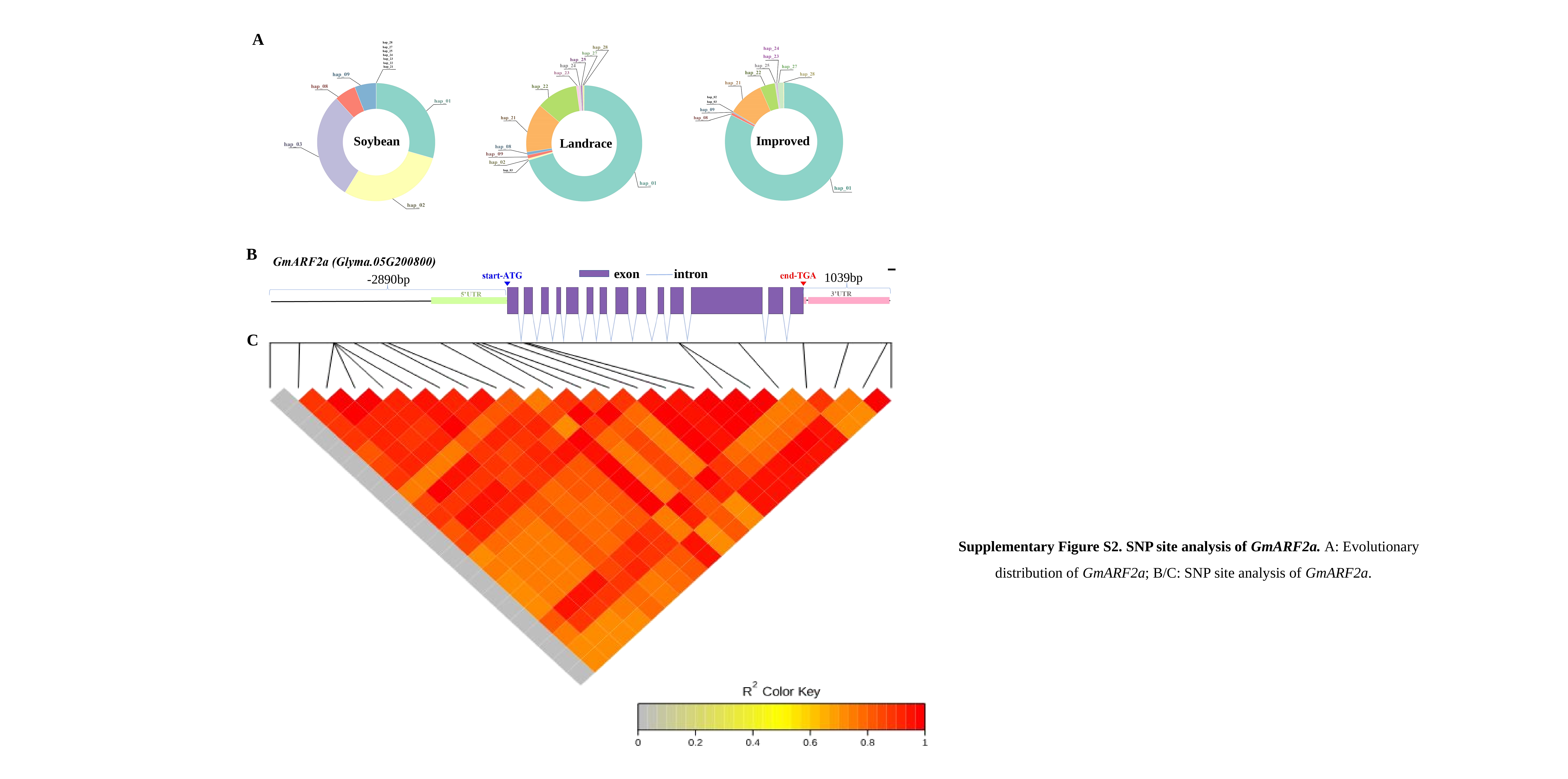

Improved
Soybean
Landrace
exon
intron
1039bp
-2890bp
A
B
C
Supplementary Figure S2. SNP site analysis of GmARF2a. A: Evolutionary distribution of GmARF2a; B/C: SNP site analysis of GmARF2a.
